# Artifacts Introduced by Sample Handling in Chemiluminescence Assays of Nitric Oxide Metabolites

**DOI:** 10.3390/antiox12091672

**Published:** 2023-08-25

**Authors:** Taiming Liu, Meijuan Zhang, Abraham Duot, George Mukosera, Hobe Schroeder, Gordon G. Power, Arlin B. Blood

**Affiliations:** 1 Department of Pediatrics, Loma Linda University School of Medicine, Loma Linda, CA 92354, USA; tliu@llu.edu (T.L.); mezhang@llu.edu (M.Z.); aduot@students.llu.edu (A.D.); 2 Lawrence D. Longo, MD Center for Perinatal Biology, Loma Linda University School of Medicine, Loma Linda, CA 92354, USA; gmukosera@students.llu.edu (G.M.); hobe.schroeder@googlemail.com (H.S.);

**Keywords:** chemiluminescence methodology, Pitfall, NO metabolites, nitrite, DNIC, heme-NO, S-nitrosothiol

## Abstract

We recently developed a combination of four chemiluminescence-based assays for selective detection of different nitric oxide (NO) metabolites, including nitrite, S-nitrosothiols (SNOs), heme-nitrosyl (heme-NO), and dinitrosyl iron complexes (DNICs). However, these NO species (NOx) may be under dynamic equilibria during sample handling, which affects the final determination made from the readout of assays. Using fetal and maternal sheep from low and high altitudes (300 and 3801 m, respectively) as models of different NOx levels and compositions, we tested the hypothesis that sample handling introduces artifacts in chemiluminescence assays of NOx. Here, we demonstrate the following: (1) room temperature placement is associated with an increase and decrease in NOx in plasma and whole blood samples, respectively; (2) snap freezing and thawing lead to the interconversion of different NOx in plasma; (3) snap freezing and homogenization in liquid nitrogen eliminate a significant fraction of NOx in the aorta of stressed animals; (4) A “stop solution” commonly used to preserve nitrite and SNOs leads to the interconversion of different NOx in blood, while deproteinization results in a significant increase in detectable NOx; (5) some reagents widely used in sample pretreatments, such as mercury chloride, acid sulfanilamide, N-ethylmaleimide, ferricyanide, and anticoagulant ethylenediaminetetraacetic acid, have unintended effects that destabilize SNO, DNICs, and/or heme-NO; (6) blood, including the residual blood clot left in the washed purge vessel, quenches the signal of nitrite when using ascorbic acid and acetic acid as the purge vessel reagent; and (7) new limitations to the four chemiluminescence-based assays. This study points out the need for re-evaluation of previous chemiluminescence measurements of NOx, and calls for special attention to be paid to sample handling, as it can introduce significant artifacts into NOx assays.

## 1. Introduction

Nitric oxide (NO) is an endogenous and short-lived (biological t_1/2_ < 2 s) gas with well-established importance in many biological processes, such as the maintenance of vascular tone, neurotransmission, and immune response [1,2]. In addition to acting as a paracrine effector, NO also plays a role as an endocrine effector probably via a range of NO metabolites (NOx), including nitrite, S-nitrosothiols (SNOs), and iron-nitrosyls (FeNO) such as heme-nitrosyl (heme-NO) and dinitrosyl iron complexes (DNICs) [3]. Nevertheless, there has long been controversy regarding which NO species subserve this function, with a core of the debate being based on the reliability of chemiluminescence-based methodologies for the detection of NOx [4,5,6].

Chemiluminescence analyzers detect NO with superior sensitivity, and have been adopted for the development of many assays for the measurements of different NOx. For example, the photolysis assay detects SNOs and N-nitroso compounds (N-NOs) through their photolytic release of NO under exposure to UV and discriminates between SNOs through their elimination via a reaction with mercury chloride (HgCl_2_) [7,8]. Other assays utilize different reagents to release NO via reactions with NOx [9,10]. Among these, the tri-iodide (I_3_^−^) reagent/assay that detects nitrite, SNOs, and heme-NO is the most popular. Since the biological samples are always a mixture of multiple NOx and a biomatrix of potential NO scavengers, various chemical pretreatments have been introduced in the I_3_^−^ assay for the purpose of the selective detection of each NO species while eliminating potential NO scavenging factors [11]. For example, a combination of chemicals known as stop or stabilization solution, or as nitrite/SNO preservation solution, is widely used to preserve the NO signal released from nitrite and SNOs in the acidic and reductive I_3_^−^ reagent. The stop solution consists of ferricyanide (oxidizes heme so as to prevent the artifactual binding of NO to the ferrous heme), N-ethylmaleimide (NEM; blocks free thiols so as to prevent the artifactual binding of NO to thiols), the detergent NP-40 (nonyl phenoxypolyethoxylethanol), and sometimes potassium cyanide (KCN) and diethylenetriamine pentaacetate (DTPA; chelates metals which degrade SNOs), and it is usually followed by methanol-mediated deproteinization [12,13]. To differentiate nitrite, SNO, and heme-NO, acid sulfanilamide (AS) and HgCl_2_ are successively added to different aliquots of the sample to selectively eliminate nitrite and convert SNO into nitrite, respectively [14,15]. The resulting differences in the NO signal were attributed to nitrite and SNO, while the NO signal left after these additions was attributed to heme-NO. However, many criticisms have been raised regarding the selectivity of this assay, and particularly that of the pretreatment reagents, since their invention [16,17,18,19,20]. Satisfactory solutions have remained elusive due to a lack of consensus on which NOx standards to use for tests, methods of comparison, and other factors, leading to significant confusion. In addition, DNICs, a photolysis and I_3_^−^-detectable NO species that has attracted increasing attention in the past decade, were omitted when pioneers established the above assays. In light of this, we have recently included DNICs as one additional target of detection and developed a combination of four chemiluminescence assays that avoids sample pretreatments [21]. Our four-assay method demonstrated an effective performance with commonly used NOx standards. However, to ensure its applicability in complex biological samples, further validation is warranted.

The hallmark of a successful NOx assay is its capability to measure NOx concentrations as they exist in vivo. Our recent measurements of fresh samples at “bedside” revealed a dynamic homeostasis of different NOx in various circulatory compartments including plasma and RBCs, the umbilical artery and vein, as well as the fetus and mother, demonstrating a rapid interconversion of these NOx in vivo [22]. However, despite our knowledge about the half-life of some specific NOx spiked into the biomatrix, whether and how rapid this interconversion of endogenous NOx occurs after sample collection remain unclear. Snap freezing samples has been commonly used in NOx investigations, with the unproven assumption that it stops all of the chemical and enzymatic reactions and thus preserves all NOx in their in vivo state. Fetal and maternal sheep from low and high altitudes (300 and 3801 m) exhibit distinct NOx levels and compositions [22]. In addition, NO metabolism has been well described in sheep in vivo, as well as in the ex vivo blood of adult and fetal sheep, and it compares well with that of humans [23,24]. Therefore, fetal and maternal sheep from low and high altitudes are valuable models for validation of a methodology under complex conditions. In the current study, utilizing samples from these animal models, we tested the effects of various sampling processes commonly employed in prevailing chemiluminescence NOx assays, including room temperature placement, snap freezing, and pretreatment with the aforementioned chemicals and methanol deproteinization. In addition, several limitations of our four-assay method are also described.

## 2. Materials and Methods

### 2.1. Chemicals

GSNO [25,26], BDNIC (binuclear DNIC), MDNIC (mononuclear DNIC) [27], and HbNO [28] were synthesized as previously described. Other chemicals were obtained from Sigma (St. Louis, MO, USA) as previously described [21,22].

### 2.2. Experimental Animals

Animal protocols were preapproved by the Institutional Animal Care and Use Committee of Loma Linda University, and were in accordance with the guidelines of the American Physiologic Society and the National Institutes of Health. For these studies, we used blood and thoracic aorta collected from sheep fetuses at about 140 days gestation and two-year-old pregnant ewes following gestation near sea level (300 m) or at an elevation of 3801 m. Their exposure to high elevation was for ~90 days before the study [29].

For each experiment, five to six sheep were used in each experimental group. For the low-altitude group (normoxic), sheep were maintained at the supplier’s ranch (Nebeker Ranch Inc.; Lancaster, CA, USA) on alfalfa pellets ad libitum. For the high-altitude group (long-term hypoxic), ewes at 50 days gestation were transported from a low altitude to the Barcroft Laboratory in the White Mountains (Bishop, CA, USA; barometric pressure −480 mmHg), where they were kept until ~135 days gestation (near term) in an outdoor sheltered pen and were fed with alfalfa pellets ad libitum. Sheep from both groups were kept in natural day–night conditions. In our previous studies [29,30] we obtained mean maternal arterial blood gas values from 12 adult sheep (same breed and age as used in the present study) while at a high altitude, which were PO_2_ = 60 ± 5 mmHg, PCO_2_ = 30.0 ± 2.5 mmHg, and pH = 7.36 ± 0.06. In contrast, normoxic low-altitude sheep had an arterial PO_2_ of 100 ± 5 mmHg, PCO_2_ = 35.2 ± 0.9 mmHg, and pH = 7.44 ± 0.1. With high-altitude exposure, the fetal arterial PO_2_ fell from 25 ± 1 to 19 ± 1 mmHg [29,30]. At 135 days gestation, ewes from both groups were transported (6 to 7 h trip) to our laboratory at Loma Linda University. Soon after the arrival at the laboratory, the ewes of the high-altitude group were instrumented with an intra-tracheal catheter for the administration of N_2_ gas and femoral arterial catheters for the measurement of blood gases, while sheep in the low-altitude group remained intact. For the high-altitude ewes, the rate of humidified N_2_ gas administered into the trachea was adjusted to maintain the PO_2_ at ~60 Torr until surgery (~5 days). The N_2_ flow was discontinued and switched to room air ~4 min before anesthesia. In both groups, the ewes were anesthetized with thiopental sodium (10 mg·kg^−1^, i.v.), and anesthesia was maintained with the inhalation of 1.5% isoflurane in 100% oxygen throughout surgery for tissue collection. From the ewes, the fetuses were delivered by hysterotomy (cesarean section). The thoracic aorta was isolated from both the fetus and ewe.

### 2.3. Sample Collection and Processing

The isolated thoracic aorta was cleaned of blood using gauze. Each aorta was cut into two segments. One segment was snap frozen in liquid nitrogen and ground into a fine powder with a mortar and pestle in liquid nitrogen within 2 h of collection. The powder was carefully dissolved in ice-cold HEPES buffer (1 g into 3 mL) and centrifuged at 3000 rpm for 15 min at 4 °C. The other segment was placed in ice-cold HEPES buffer and, within 2 h of collection, homogenized using a rotor-stator homogenizer (TissueRuptor, Qiagen Inc.; Hilden, Germany) in ice-cold HEPES buffer (1 g in 5 mL) and then centrifuged at 3000 rpm for 15 min at 4 °C. The supernatant of each segment was snap frozen in an Eppendorf tube and stored at −80 °C for 2 to 5 days until assayed.

Blood samples were collected with heparinized syringes. In both the high- and low-altitude groups, the adult venous whole blood (AVWB) was collected from the jugular vein of the ewe right before anesthesia (high-altitude ewes breathed room air for ~3 min). Fetal whole blood was collected from the umbilical cord artery (UAWB; deoxygenated blood from the fetus) and vein (UVWB; oxygenated blood from the placenta) after cesarean section and before cord clamping. Plasma was separated from the red blood cells immediately after blood collection by centrifugation at 10,000 rpm for 30 s. To minimize changes in the metabolism during sample handling, one aliquot of each of the whole blood and plasma samples was measured within 1 to 4 min of blood collection (named intact as the control). Other aliquots were treated as described in the figure legends. As previously described [31], the stop solution used in the current study consisted of 0.8 M ferricyanide (K_3_[Fe(CN)_6_]), 10 mM NEM, and 1% NP-40 (to lyse lipid membranes).

### 2.4. Analytical Methods

Electron paramagnetic resonance (EPR) signals were recorded at 150 K using a Bruker X-Band EMX Plus EPR spectrometer with a high-sensitivity cavity as previously described [27]. The EPR settings were as follows: microwave power of 20 mW, microwave frequency of 9.31 GHz, attenuator of 10 dB, modulation amplitude of 5 G, modulation frequency of 100 kHz, time constant of 10.24 ms, conversion time of 40.96 ms, harmonic of 1, and scan number of 2.

NO metabolite (NOx) levels were measured by four different assays with the chemiluminescence nitric oxide analyzer (280i, Sievers, Boulder, CO, USA) as previously described [21]. In each assay, the sample reacts to the release of NO in the purge vessel of the analyzer using appropriate reagents. The released NO gas is carried by a stream of argon to the detector, where it reacts with ozone to produce a chemiluminescence signal proportional to the quantity measured. The reagents used in the four assays include: triiodide (I_3_^−^; measures nitrite, SNO, DNIC, and heme-NO), ferricyanide + ascorbic acid (K_3_[Fe(CN)_6_]/AcOH; measures nitrite, DNIC, and heme-NO), ferricyanide + PBS (K_3_[Fe(CN)_6_]/PBS; measures DNIC and heme-NO), and ascorbic acid + acetic acid (VitC/AcOH; measures nitrite and heme-NO). The purge vessel was washed at least once and the reagent was replaced with fresh reagent after each injection.

### 2.5. Statistics

Average values are presented as mean ± SEM in the text and figures. One-way ANOVA, two-way ANOVA, linear regression, and a t-test were used, as indicated in the figure legends. Repeated measures were used when applicable. Statistical analyses were performed with Prism, v8.4.0 (Graphpad Software, La Jolla, CA, USA), with significance accepted when *p* < 0.05.

## 3. Results

### 3.1. Effects of Room Temperature Placement on Plasma and Blood NOx

**Figure 1:**
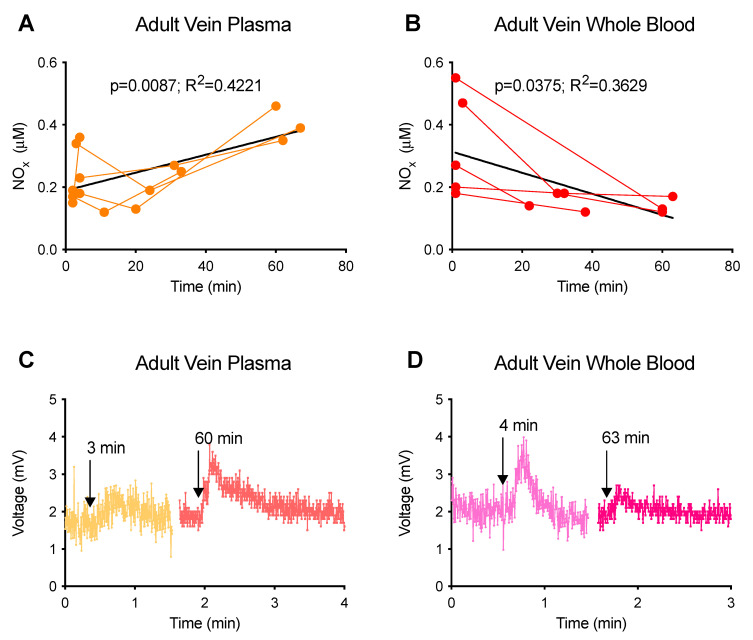
Effects of incubation at room temperature on NOx signals. *n* ≥ 5. Samples were kept in the dark at room temperature and measured using I_3_^−^ as the purge vessel reagent at different time points after collection. (**A**,**B**) Plot of the time dependence of NOx levels. Data points from each individual animal are connected by lines. Black lines show the linear regression with slopes significantly different from zero. (**C**,**D**) Representative NOx signal traces. Two trimmed traces of injections were clipped and lined up on one continuous *x*-axis for better comparison. Arrows indicate the injections made at different time points after blood sample collection. (**A**,**C**) Adult vein plasma (AVP). (**B**,**D**) Adult vein whole blood (AVWB). AVP and AVWB were from healthy low-altitude sheep. Between injections, the purge vessel was washed at least once with DI-water and refilled with fresh reaction reagent.

The question has often been raised about how long samples can be kept at room temperature before further treatment or analysis. Through the linear regression of the injections made in I_3_^−^ at different timepoints after sample collection, our experiments demonstrated that placing the samples at room temperature for 1 h resulted in a time-dependent increase (~100%) in NOx in adult venous plasma and a decrease (~60%) in adult venous whole blood (Figure 1).

### 3.2. Effects of Liquid Nitrogen Snap Freezing on Plasma NOx

**Figure 2:**
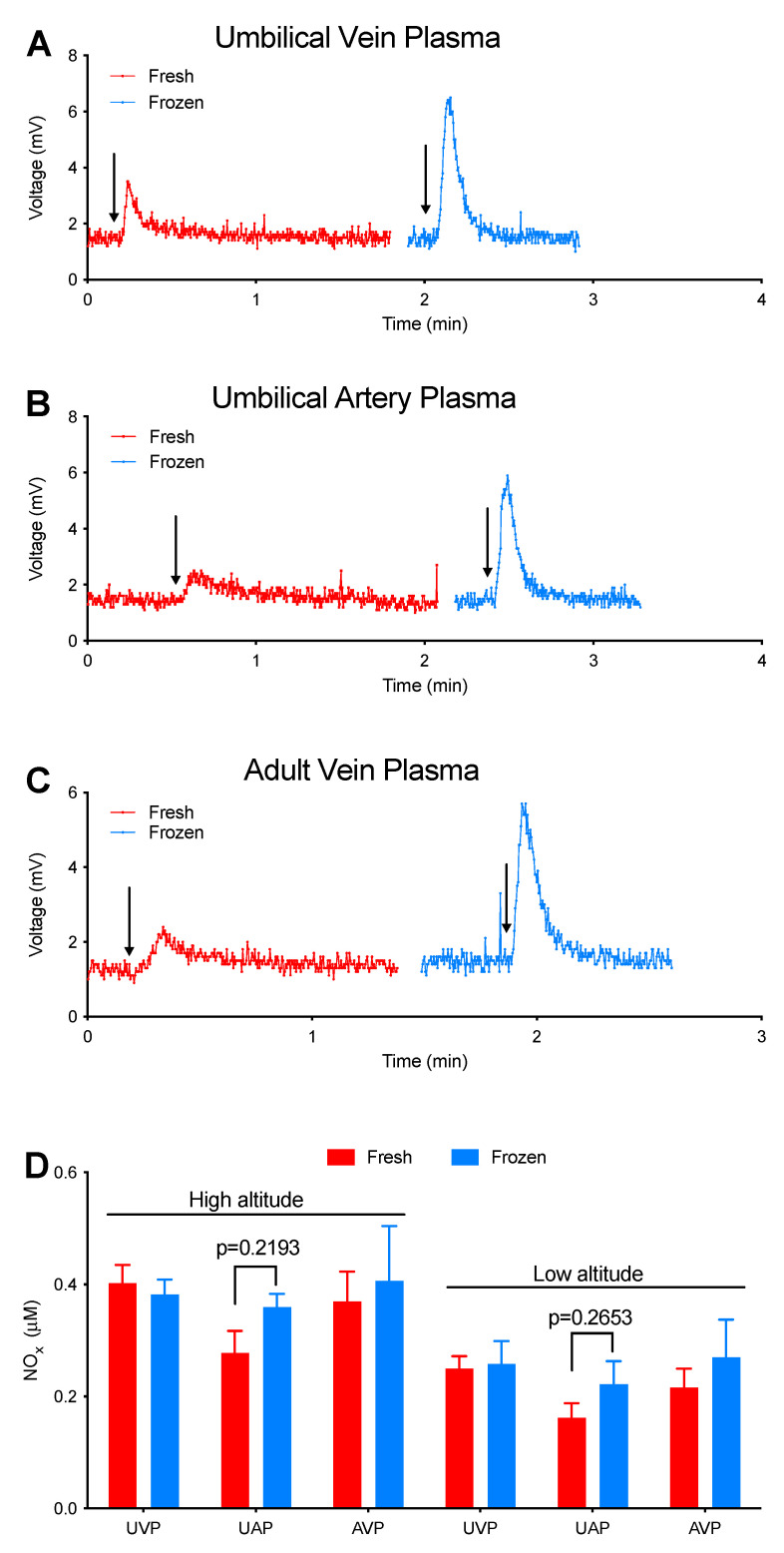
Effects of snap freezing in liquid nitrogen on the NOx signals of plasma from high- and low-altitude sheep. *n* ≥ 5. NOx were measured using I_3_^−^ as the purge vessel reagent. (**A**,**C**) Representative NO signal of UVP (**A**), UAP (**B**), and AVP (**C**). Each plasma sample was divided into two aliquots: the first (red) was intact and measured within 4 min of blood collection; the second (blue) one was snap frozen in liquid nitrogen for 30 min and then thawed in hand for measurement within 4 min. All traces (**A**,**C**) were from one healthy high-altitude sheep. Arrows represent injections. (**D**) Summary of the effects of snap freezing on NOx quantities as determined by measurement of the area under the peak. Despite significant qualitative differences in the shapes of NOx peaks between fresh and frozen samples from both high- and low-altitude ewes, no significant difference was detected in the quantities by paired *t*-tests. UVP: umbilical vein plasma; UAP: umbilical artery plasma; AVP: adult vein plasma.

We next tested the effects of liquid nitrogen snap freezing on I_3_^−^ measurements of plasma NOx. As shown in Figure 2, fresh plasma samples resulted in broad and short signal peaks similar to that of FeNO, whereas the parallel snap-frozen samples resulted in peaks as sharp and high as those of nitrite and SNOs, suggesting that snap freezing leads to the interconversion of different I_3_^−^-detectable NOx in plasma. Despite these alterations in NO species, the quantities of NOx were not significantly affected. It is worth noting that all peaks were integrated manually, with the selected peak width range being as wide as possible. When the signal returns to the baseline, the increase in the time range of selection has little effect on the area of the peaks.

### 3.3. Liquid Nitrogen Treatments Decrease NOx in Aorta of Stressed Animals

**Figure 3:**
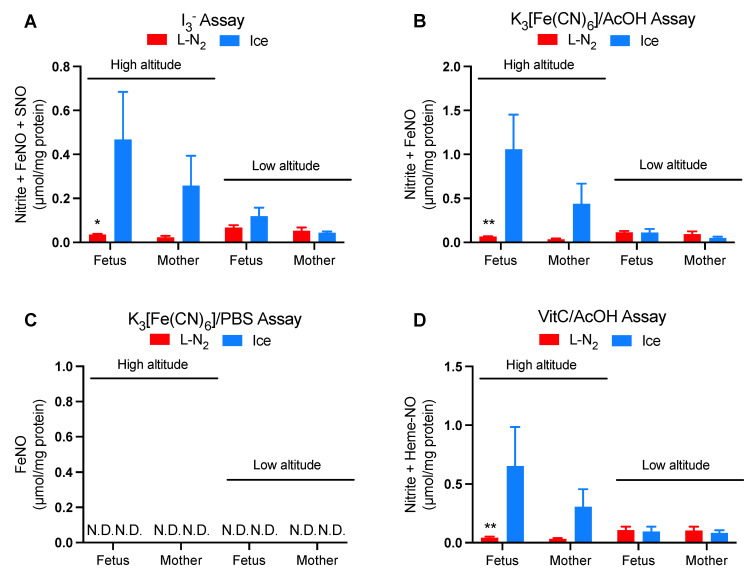
Effects of methods of homogenization on NOx levels in aorta homogenates. *n* ≥ 5. Thoracic aortas were homogenized in either liquid nitrogen (L-N_2_) or ice-cold HEPES buffer (Ice). NOx were measured by chemiluminescence NO analyzer with different purge vessel reagents. (**A**) I_3_^−^. (**B**) K_3_[Fe(CN)_6_]/AcOH. (**C**) K_3_[Fe(CN)_6_]/PBS. (**D**) VitC/AcOH (see the Materials and Methods section or reference #21 for explanation of which NOx metabolites are detected by each reagent). Measurements were normalized to protein concentrations in the homogenates. N.D.: not detectable. Two-way ANOVA with Sidak’s post hoc tests. * for *p* < 0.05 and ** for *p* < 0.01 for L-N_2_ vs. Ice. Results of the ice-cold HEPES buffer group were recently reported elsewhere [22].

We also tested for the effects of liquid nitrogen treatments (snap freezing and homogenization) on NOx in aortic tissues. In samples from high-altitude animals, compared with homogenization in ice-cold HEPES, homogenization in liquid nitrogen resulted in lower NOx in the fetal aorta as measured by I_3_^−^, K_3_[Fe(CN)_6_]/AcOH, and K_3_[Fe(CN)_6_]/AcOH assays (Figure 3A,B,D). Although significance was not reached due to large intragroup fluctuations, a similar trend of NOx alteration was also observed in the aortas of high-altitude pregnant ewes. At low altitude, no significant difference was found between the two methods of homogenization (Figure 3A–D). Given that fetuses are more stressed than the mothers and that high-altitude superimposes stresses on fetuses, the above results suggest that the decrease in aorta NOx caused by liquid nitrogen treatments is associated with hypoxic stress in animals. For reasons unknown, no NOx were detected by the K_3_[Fe(CN)_6_]/PBS assay (Figure 3C), demonstrating the absence of both heme-NO and DNICs.

### 3.4. Effects of Stop Solution and Deproteinization on Whole Blood NOx

**Figure 4:**
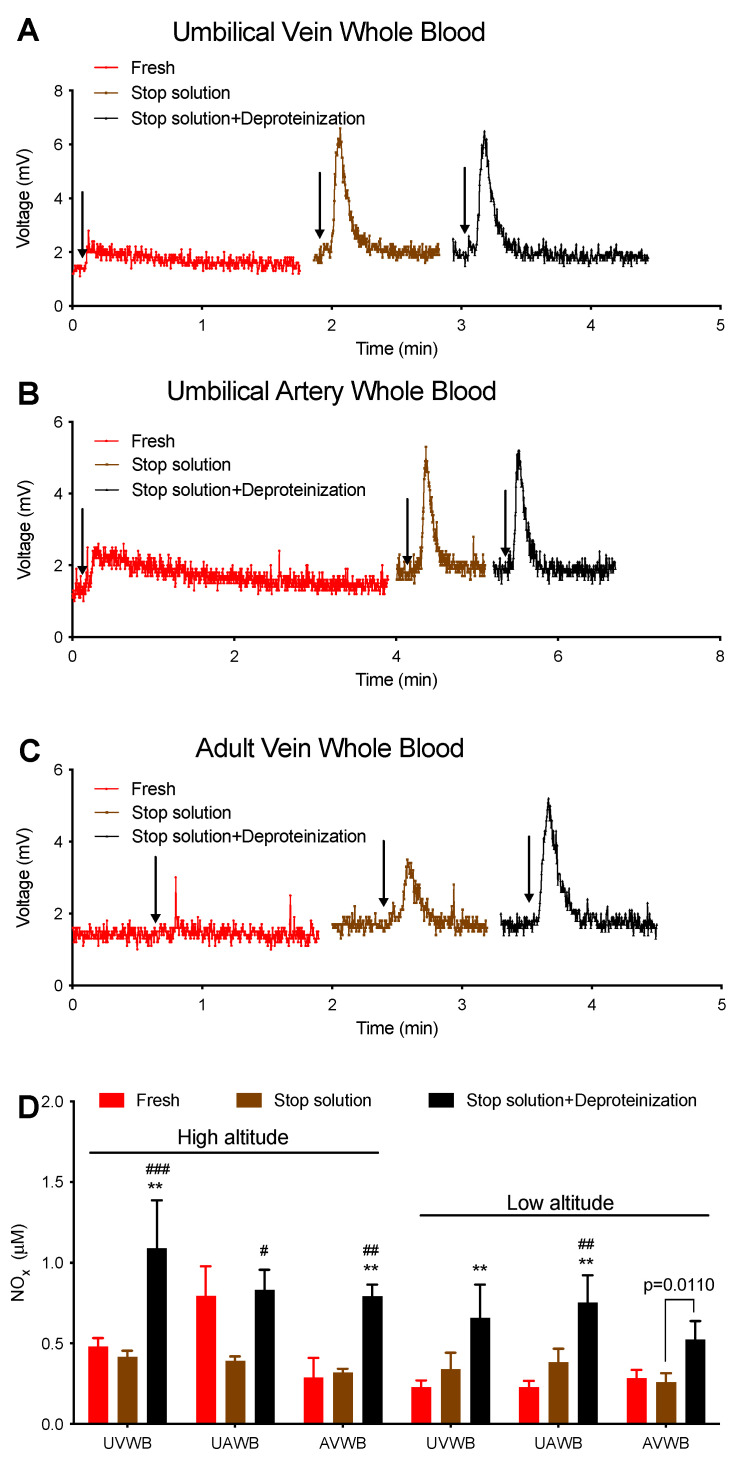
Effects of stop solution and deproteinization on the NOx signals of whole blood samples from high- and low-altitude sheep. *n* ≥ 5. Samples were measured using I_3_^−^ as the purge vessel reagent. (**A**,**C**) Representative NOx signal of UVWB (**A**), UAWB (**B**), and AVWB (**C**). Whole blood was divided into three aliquots: the first one (red) was left intact and measured within 4 min of blood collection; the second one (brown) was measured after mixing sample with stop solution at a volume ratio of 5:1; the third one (black) was prepared by mixing the second one with cold methanol at a volume ratio of 1:1 and then centrifugation (deproteinization) at 10,000 rpm for 30 s for supernatant. All traces (**A**,**C**) were from one representative high-altitude sheep. Arrows represent injections. Parallel injections of stop solution and methanol per se did not result in any NO signal in the assay. (**D**) Summary of the effects of stop solution and deproteinization on NOx quantities after correction for dilution factors. Two-way ANOVA. ** for comparison with control; # for comparison with the stop solution. Single symbol for *p* < 0.05; double symbols (** and ##) for *p* < 0.01; triple symbols (###) for *p* < 0.001. *p*-value in (**D**) for paired *t*-test.

The above failure of snap freezing to stabilize NOx led us to test for the effects of the commonly used stop solution and deproteinization on whole blood NOx. Although the calculated concentrations of NOx were not altered by the stop solution treatment, the NOx peaks obtained from the untreated fresh whole blood samples, as measured by I_3_^−^, were short and had long tails, whereas peaks obtained after treatment with the stop solution were tall and narrow (Figure 4). The former signal resembles FeNO, and especially heme-NO as it has a longer tail than DNICs, while the latter signal is similar to that of nitrite or SNO, suggesting a possible conversion of heme-NO into nitrite or SNO by the treatment with the stop solution. Further treatment with methanol for deproteinization did not alter the shape of the peaks; however, it resulted in a two-fold increase in the quantity of NOx.

### 3.5. Effects of Several Reagents on the Stability of GSNO, BDNIC, MDNIC, and Heme-NO

The unexpected effects of the stop solution observed above led us to test the effects of widely used sample pretreatment chemicals on the stability of several representative NOx standards including GSNO, BDNIC, MDNIC, and heme-NO. Table 1 summarizes the results from the EPR measurements and chemiluminescence assays using an HEPES buffer as the purge vessel reagent (see Supplementary Materials, Figures S1–S3 for details).

As shown in Table 1, all of these reagents unintentionally release NO from one or more of the tested NOx species. For example, HgCl_2_, which was used to selectively eliminate SNOs in both I_3_^−^ and the less popular photolysis chemiluminescence assay, actually also released NO from DNICs. Acid sulfanilamide (AS), which was used to selectively eliminate nitrite, released NO from both DNICs and heme-NO. HgCl_2_, together with AS, released NO from all tested NOx. NEM, which was used to prevent the artifactual formation of SNOs via the blockage of free thiol, somehow eliminated the EPR signal of MDNIC, although it did not seem to release free NO from DNICs. Ferricyanide, which was used to oxidize ferrous heme into ferric heme to avoid the artifactual binding of NO to the ferrous heme in the purge vessel, released NO from all tested NOx. Ethylenediaminetetraacetic acid (EDTA; an anticoagulant in blood sampling tubes) released NO from both SNO and DNICs. Additionally, DTPA, a chelator widely used in experiments with SNOs, eliminated the EPR signal of MDNIC, while another chelator, DFO, had no such effect.

### 3.6. Effects of Blood on NOx Measurement with VitC/AcOH Assay

**Figure 5:**
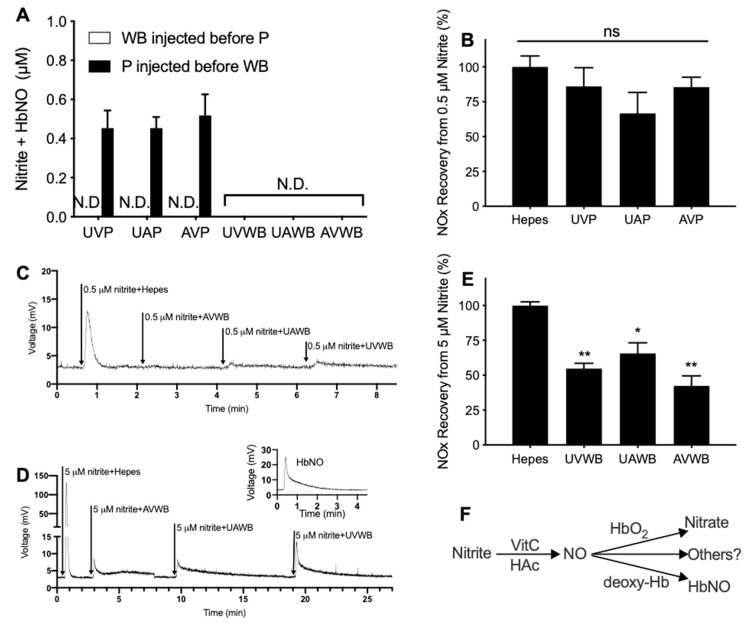
Effects of blood on NOx measurements with VitC/AcOH assay, which detects nitrite and heme-NO [21]. *n* ≥ 5. Samples were measured using VitC/AcOH as the purge vessel reagent. (**A**) NOx was not detected in whole blood no matter if it was injected before or after plasma, whereas NOx was detected in plasma only when it was injected before whole blood. Between injections, the purge vessel was washed at least once with DI-water and then filled with fresh reaction reagent. However, trace blood clots could still be observed on the wall of the purge vessel. (**B**) Plasma did not significantly quench the NOx signal of the spiked 0.5 μM of nitrite. (**C**,**D**) Representative traces showing that whole blood significantly quenched the NOx signal when spiked with 0.5 μM (**C**) and 5 μM (**D**) of nitrite. Inset in (**D**) is a representative trace of iron-nitrosyl hemoglobin (HbNO). (**E**) Recovery of NOx from whole blood spiked with 5 μM of nitrite. Nitrite was spiked into HEPES (100%), plasma, or blood, and measured within 4 min. (**F**) Proposed diagram for the effects of blood on nitrite measurements with VitC/AcOH assay. *: for *p <* 0.05, **: for *p* < 0.01, ns for no significant difference, One-way ANOVA versus HEPES. P = plasma, WB = whole blood, UVP = umbilical venous plasma, UAP = umbilical arterial plasma, AVP = adult venous plasma, UVWB = umbilical venous whole blood, UAWB = umbilical artery whole blood, AVWB = adult venous whole blood.

The VitC/AcOH (ascorbic acid + acetic acid) assay is a measurement of nitrite and heme-NO. In this assay, we surprisingly found that blood samples had significant carryover effects even if the purge vessel was extensively rinsed with water. When plasma was injected before whole blood, it resulted in a NOx signal of about 0.5 μM, while injection after whole blood gave no signal at all (Figure 5A). In addition, the VitC/AcOH assay did not detect any nitrite or heme-NO in the whole blood samples, which contradicts the common knowledge that blood typically contains approximately 0.1 to 0.5 μM of nitrite and that heme effectively scavenges NO released from nitrite under acidic conditions, leading to heme-NO production. These results indicate that blood, including the residual blood clot left in the washed purge vessel, may diminish the signal of both nitrite and heme-NO.

Consequently, with the use of purge vessels extensively washed with NaOH (removes proteins) and water, we tested the NOx recovery of 0.5 μM of nitrite spiked into plasma and whole blood in the VitC/AcOH assay. Compared to the control (HEPES buffer), the recovery of NOx from 0.5 μM of nitrite spiked into plasma was slightly decreased (no significance observed), possibly because of trace hemolysis. However, almost no NOx was recovered when 0.5 μM was spiked into blood (Figure 5B,C). Furthermore, when as high as 5 μM of nitrite was spiked into whole blood, the immediate measurement of this mixture resulted in short peaks with long tails, resembling heme-NO (Figure 5D). Quantification of these signals revealed that blood quenched up to 60% of the NOx signals compared with the HEPES buffer when 5 μM of nitrite was spiked (Figure 5E). These results suggest that a significant fraction of NOx was turned into something undetectable in the assay. The diagram illustrating the proposed effects of blood on nitrite measurements in the VitC/AcOH assay is given in Figure 5F.

## 4. Discussion

The current study demonstrates the presence of significant artifacts in prevailing chemiluminescence NOx assays introduced by commonly applied sample handling processes, including snap freezing and various pretreatment chemicals such as acid sulfanilamide and stop solution, suggesting that a re-evaluation of previous measurements of NOx is needed. Additionally, we also identified several defects in our recently developed four-assay method [21] for the selective determination of different NOx species, and we describe here contemporary perspectives and technical challenges.

### 4.1. Artifacts Introduced by Liquid Nitrogen Treatments of Samples

I_3_^−^-detectable NOx time-dependently increase (~100%) in adult venous plasma and decrease (~60%) in adult venous whole blood during incubation for 1 h at room temperature (Figure 1), indicating that blood samples should be assayed as soon as possible after collection. Snap freezing in liquid nitrogen is routinely used for the treatment of plasma samples in many labs. However, our parallel measurements of fresh and snap-frozen plasma samples demonstrate that snap freezing results in the rapid interconversion of different I_3_^−^-detectable NOx, possibly converting FeNO into nitrite and/or SNOs (Figure 2). These results indicate that snap freezing might have significantly overestimated and underestimated the levels of nitrite and FeNO, respectively, in previous plasma measurements.

Furthermore, our comparison of homogenization in liquid nitrogen and ice-cold HEPES buffer surprisingly demonstrates that liquid nitrogen treatments lead to decreases in NOx measured by three different assays in the aorta from stressed animals [22] (Figure 3). Although it remains unclear if these decreases result from the snap-freezing process, the subsequent homogenization in liquid nitrogen, or both, these results raise the possibility that previous measurements of liquid-nitrogen-treated tissues from stressed animal models may have underestimated the NOx levels. Together, these findings suggest that liquid nitrogen treatments (including the thawing process), a procedure essential for research and biobanks, introduce significant alterations in the composition and levels of NOx.

The mechanisms underlying the liquid-nitrogen-mediated NOx alterations were not investigated. The liquid-nitrogen-mediated NOx alterations may be related to the denaturation of proteins, the alteration of pH and pKa, changes in redox status and oxygen availability, and others associated with snap freezing [32,33,34]. For example, it has been proposed that there is a considerable amount of NO in the hydrophobic zone of albumin [35]. Denaturation and other effects on albumin caused by snap freezing may lead to the release and conversion of this fraction of NOx. Additionally, there is increasing evidence that nitrogen is not always inert and may participate in some reactions [36,37].

### 4.2. Artifacts Introduced by Chemical Pretreatments of Samples

Similar to the liquid nitrogen treatments, the stop solution and the subsequent methanol deproteinization also result in significant alterations in the composition and levels of NOx. Consistent with our recent observations that the placenta secretes FeNO, and more specifically, probably hemoglobin-NO, into the umbilical blood [22,38,39], the shape of the NOx signal from the injection of fresh whole blood into I_3_^−^ resembles that of heme-NO. However, the shape of the NOx signal after incubation with the stop solution is similar to that of nitrite or SNO (Figure 4), suggesting that the conversion of heme-NO into nitrite or SNO is induced by the stop solution. This conversion can be, at least, partially attributed to the destruction of heme-NO by ferricyanide (Table 1 and Figure S2). It is important to highlight that the inclusion of ferricyanide in the stop solution serves to prevent the artificial formation of heme-NO from endogenous nitrite under the acidic condition of the I_3_^−^ assay. It has been widely assumed that nitrite is a primary form of NOx in whole blood samples. Nevertheless, our compelling demonstration using the K_3_[Fe(CN)_6_]/PBS assay reveals that the predominant endogenous NOx in the tested whole blood samples is FeNO, not nitrite [22,38,39].

Unexpectedly, both NEM and DTPA, two other reagents in the stop solution, appear to eliminate DNICs, as manifested by the disappearance of the EPR signal of MDNIC in the presence of NEM or DTPA (Table 1 and Figure S3). These results indicate that the stop solution, also referred to as nitrite/SNO-preserving solution, may artifactually disrupt heme-NO and DNICs and lead to an overestimation of nitrite/SNO levels in blood samples. Further deproteinization of the blood sample by methanol, which does not result in any NO signal per se, results in a doubling of the NOx level. These results are consistent with previous observations using the Griess assay, whereby deproteinization with methanol, ethanol, and diethyl ether led to higher NOx values in serum. This phenomenon was attributed to the interferences between an organic solvent and Griess reagents [40,41]. Although it remains unknown whether the increase in NOx can be attributed to NO released from the hydrophobic zone of proteins or other sources, this artifact introduced by methanol deproteinization explains why surprisingly high levels of nitrite are observed in red blood cells treated with methanol [31].

In addition to the stop solution, as demonstrated by others and our own group [21,42], HgCl_2_ and/or AS also compromise the measurements of NOx as evidenced by the release of NO from DNICs by HgCl_2_, and from both DNICs and heme-NO by AS (Table 1 and Figures S1 and S2). These effects of HgCl_2_ and AS are not unexpected since DNICs were not adequately considered during the early validations of the chemiluminescence methodology for measurements of NOx [14,15]. Although the endogenous levels of DNICs in biological samples are yet to be determined, there is increasing evidence demonstrating the prevalence of DNICs as a form of NO signaling in biology [43,44]. With this understanding, the aforementioned artifacts of HgCl_2_ and AS on DNICs must be taken into account.

We also observed that the anticoagulant EDTA releases NO from both GSNO and DNICs, suggesting potential artifacts introduced by some blood sampling tubes. It is important to note that, in the current study, except for EDTA which is used at a concentration 4-fold higher than that of a normal application, most of the aforementioned chemicals are used at concentrations much lower than those widely applied. This suggests that their artifactual effects in previous applications should be more pronounced than what was observed here.

### 4.3. Limitations of Our Four-Assay Method

In this study, several limitations were identified regarding our four-assay method, despite avoiding the use of the above chemicals that introduce artifacts in NOx measurements. First, in the VitC/AcOH assay, which measures nitrite and heme-NO, the presence of hemoglobin diminishes the detection of endogenous nitrite (Figure 5). This finding is consistent with our previous negative measurements of blood NOx with the VitC/AcOH assay [21]. Recovery experiments with increased levels of nitrite spiked into blood demonstrate that nitrite is converted into both heme-NO and some undetectable products. It is possible that, via the reaction with AcOH, nitrite rapidly releases NO in the purge vessel, which is then captured by deoxy-Hb and HbO_2_ to generate heme-NO and the undetectable nitrate, respectively. Nevertheless, because the residual clot of blood, which probably is fully deoxygenated, left within the washed purge vessel is able to completely diminish signals of endogenous NOx, other pathways of NO scavenging must exist. Notably, VitC (sodium ascorbate) at an applied high concentration may generate superoxide, while heme iron can significantly facilitate redox cycling [45,46]. It is possible that the redox condition of the VitC/AcOH reagent favors the generation of peroxynitrite and/or other nitrogen oxides that are undetectable by chemiluminescence (Figure 5F). These results suggest that the VitC/AcOH assay is not suitable for blood and possibly other samples rich in metals or metalloproteins. Further investigation into the mechanisms underlying the NOx elimination phenomenon is needed.

Second, ferricyanide unexpectedly releases NO from GSNO (Table 1 and Figure S1). These results contradict our recent findings during the establishment of the ferricyanide/PBS assay for the selective detection of FeNO in the presence of GSNO and nitrite. GSNO is known to release NO in the presence of heavy metals [47]. The results presented in Figure S1 of the current study were actually generated over 8 years ago with an old batch of ferricyanide, which might have contained FeCl_2_, a chemical used in the production of ferricyanide. It is likely that this artifact results from Fe^2+^/Fe^3+^ contamination of ferricyanide. These results raise awareness of the need for not only the re-evaluation of previous results of NOx detection in the presence of ferricyanide, but also the evaluation of the purity of ferricyanide in future NOx assays.

Third, the NO mass balance cannot be achieved with the four-assay method (Figure 4). In our recent work with the four-assay method, as well as the current study, the NOx levels measured by the K_3_[Fe(CN)_6_]/AcOH assay were often about double those measured by the I_3_^−^ assay, which additionally detects SNOs. This issue occurs with plasma, whole blood [22], and also artery homogenates (Figure 4). These results indicate that the I_3_^−^ assay may underestimate the levels of SNO and/or FeNO, and/or that the K_3_[Fe(CN)_6_]/AcOH assay may detect other NOx species in addition to nitrite and FeNO. Consistent with our findings, there has been much evidence suggesting that the I_3_^−^ assay underestimates different NOx species [20]. For instance, in the I_3_^−^ assay, the yield of NO production from heme-NO is only 81% of the theoretical calculation [14], and it could be increased by the addition of ferricyanide [19]. Additionally, while low-molecular-weight SNOs commonly used as standards have yields of 100%, the high-molecular-weight SNOs, which are more abundant in biological samples, have lower yields, as demonstrated by the 78% yield of S-nitroso-albumin in I_3_^−^ [14]. On the other hand, there is increasing evidence showing off-target detection of NOx species in the K_3_[Fe(CN)_6_]/AcOH assay. For example, we have demonstrated that deferoxamine, a hydroxamic acid used as a chelator in basic research and clinics, is capable of releasing NO in the presence of ferricyanide but not I_3_^−^ [48]. Additionally, N-acetylcysteine (NAC) and metformin (not shown) are also able to release trace NO from the reaction with ferricyanide (Figure S4). These results suggest that ferricyanide may unexpectedly release NO from a wide range of different chemical structures. It is possible that some of these chemical structures exist in biological samples, and thus contribute to the higher levels of NOx measured by the K_3_[Fe(CN)_6_]/AcOH assay than that by the I_3_^−^ assay.

## 5. Perspectives

Given that artifacts in the NOx measurements can be introduced by the incubation of samples at room temperature or by snap freezing, we recommend immediate measurements for biological NOx samples. When this is not possible, the use of liquid nitrogen snap freezing and its potential effects, as demonstrated herein, should be assessed and highlighted. All previously described chemical pretreatments are accompanied by artifacts and should therefore be avoided when possible, or only used with the full disclosure of their potential limitations. With the exclusion of chemical pretreatments and the inclusion of DNICs as an additional target of NOx detection, our four-assay method [21] may be more reliable than previous chemiluminescence NOx assays. Nevertheless, this methodology is still based on the use of multiple chemical reagents which are inevitably accompanied by unidentified side effects. Therefore, the further evaluation and development of the methodology, especially its use in complex biological samples and pathophysiological models, are warranted.

Notably, while there is little doubt that NO-modified proteins play a crucial role in a wide range of biological processes, as well as in the storage and regulation of NO bioactivity [3], major advances in our understanding of these aspects of NO signaling can only be achieved through the discovery of innovative methods for detecting and quantifying these individual macromolecules rather than relying on overall lumped categories of NOx species. In light of this, we advocate for the development of novel methodologies. Ideally, targeted and non-targeted methodologies of chromatography followed by mass spectrometry, which is capable of qualifying and quantitating known and unknown NOx species, should both be developed. However, to achieve these goals, there are several technical challenges that must be addressed. These challenges include maintaining the stability of NOx during sample collection and homogenization, as well as during the separation/enrichment of NOx. Moreover, ensuring the stability of NOx in an ion source and mitigating ion suppression in NOx are also critical considerations. Additionally, if derivatization is employed, the chemical selectivity of reagents as encountered in the GC-MS method [46] needs to be carefully addressed.

## 6. Conclusions

In summary, our findings reveal that placing samples at room temperature, snap freezing, chemical pretreatment, and deproteinization all can introduce inaccuracies in the prevailing chemiluminescence-based NOx assays. Moreover, our four-assay method displays certain limitations. Although this study might prompt more inquiries than it resolves, it underscores the necessity to re-evaluate past chemiluminescence-derived NOx measurements and emphasizes the importance of meticulously addressing sample handling to avoid substantial artifacts in NOx assays.

## Figures and Tables

**Table 1:** Effects of several chemicals widely used in processing of NOx samples on the stability of GSNO, BDNIC, MDNIC, and heme-NO (HbNO). *n* = 3.

Reagents	GSNO	BDNIC	MDNIC	Heme-NO
HgCl_2_ (2.5 mM)	+	+	+	−
AS (0.125% *w*/*v*)	−	+	+	+
HgCl_2_ + AS	+	+	+	+
NEM (100 μM)	−	?	?	−
K_3_[Fe(CN)_6_] (0.5 mM)	+	+	+	+
EDTA (25 mM)	+	+	+	−

Note: “+” = release NO; “−” = no effects; “?” = unclear effects. For details, see Supplementary Materials, Figures S1–S3.

## Data Availability

Data are contained within the article.

## Supplementary Materials

The following supporting information can be downloaded at: https://www.mdpi.com/article/10.3390/antiox12091672/s1, Figure S1: Effects of several chemicals, commonly used in processing of NOx samples, on the stability of GSNO, BDNIC, and MDNIC.; Figure S2: Effects of several chemicals commonly used in processing of NOx samples on the stability of heme-NO (HbNO). Figure S3: Representative EPR traces showing effects of DTPA, NEM, and DFO on MDNIC signals. Figure S4: Ferricyanide releases NO from N-acetylcysteine (NAC).
