# Supplementary material for: Artifacts Introduced by Sample Handling in Chemiluminescence Assays of Nitric Oxide Metabolites
Source: Antioxidants (Basel). 2023 Aug 25;12(9):1672. doi: 10.3390/antiox12091672 (PMC10525973; doi:10.3390/antiox12091672)
Supplement: Supplementary file 1 [file antioxidants-12-01672-s001.zip › antioxidants-2558484-supplementary.pdf]

Supplementary Materials

Artifacts introduced by sample handling in chemiluminescence assays of nitric oxide metabolites

Taiming Liu[1], Meijuan Zhang[1], Abraham Duot[1], George Mukosera[2], Hobe Schroeder[2], Lubo Zhang[2], Gordon G. Power[2†], Arlin B. Blood[2*]

[1]Department of Pediatrics, Loma Linda University School of Medicine, Loma Linda, CA;
[2]Lawrence D. Longo, MD Center for Perinatal Biology, Loma Linda University School of Medicine, Loma Linda, CA.
†Passed away.

[*]Correspondence to: Arlin Blood. PhD, Lawrence D. Longo, MD Center for Perinatal Biology, Loma Linda University School of Medicine, Loma Linda, CA.
Email: abblood@llu.edu

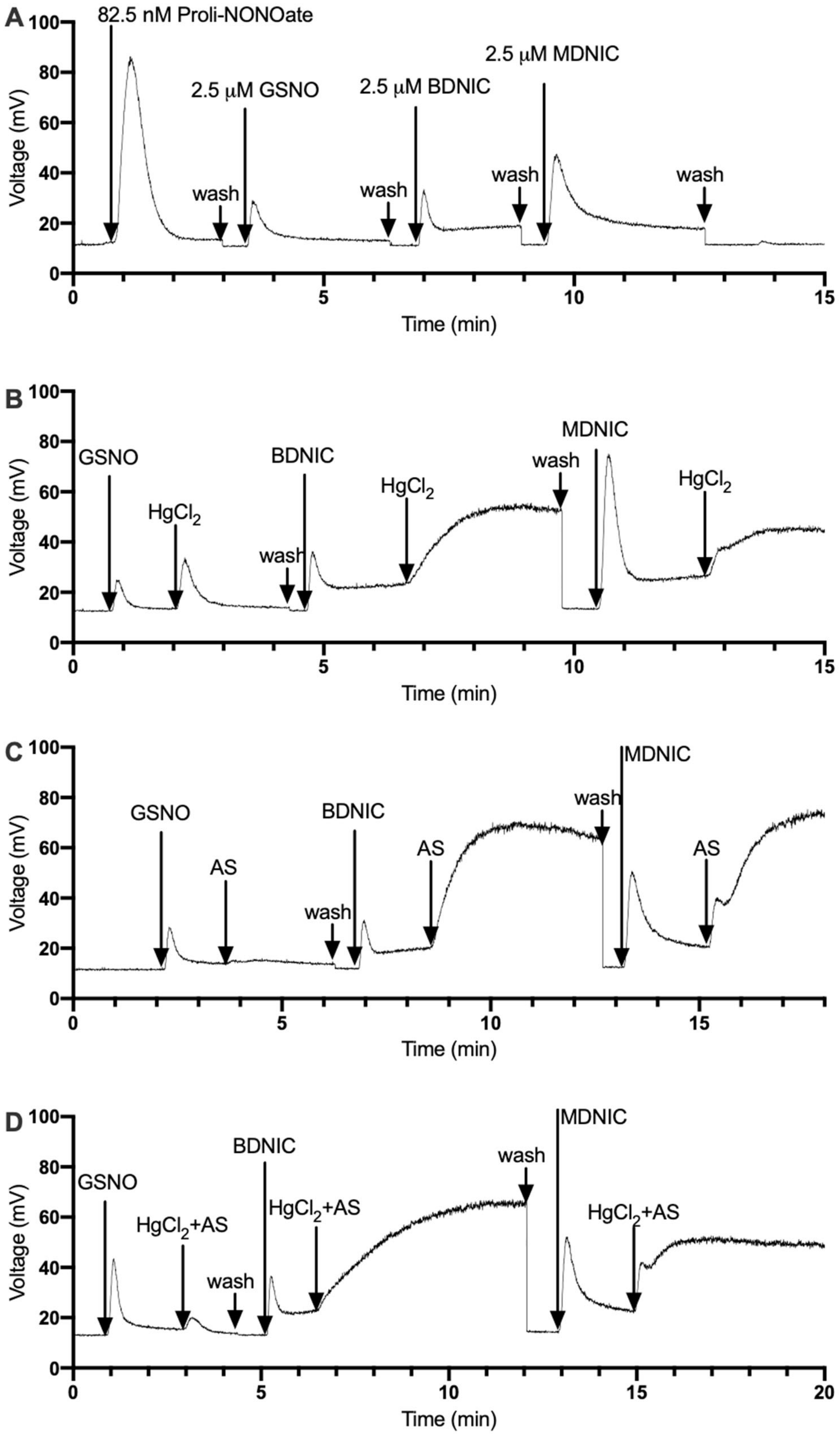
A
82.5 nM Proli-NONOate
2.5 μM GSNO
2.5 μM BDNIC
2.5 μM MDNIC
wash
wash
wash
wash
Voltage (mV)
Time (min)
B
GSNO
HgCl2
wash
BDNIC
HgCl2
wash
MDNIC
HgCl2
Voltage (mV)
Time (min)
C
GSNO
AS
wash
BDNIC
AS
wash
MDNIC
AS
Voltage (mV)
Time (min)
D
GSNO
HgCl2+AS
wash
BDNIC
HgCl2+AS
wash
MDNIC
HgCl2+AS
Voltage (mV)
Time (min)

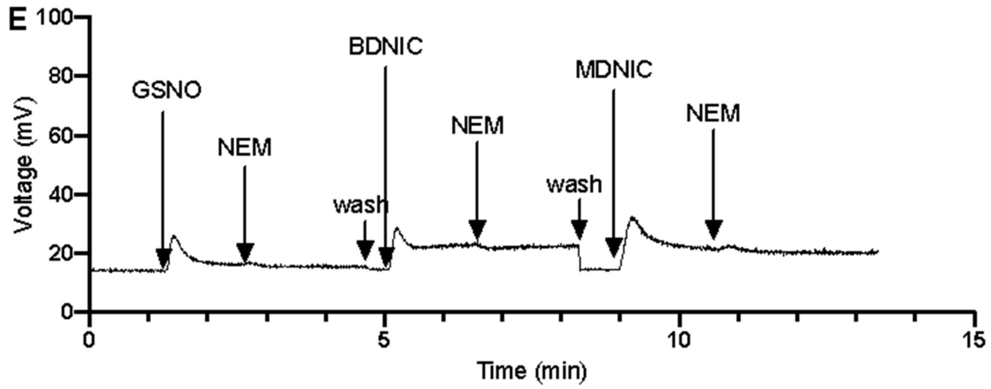
E
GSNO
NEM
wash
BDNIC
NEM
wash
MDNIC
NEM
Voltage (mV)
Time (min)

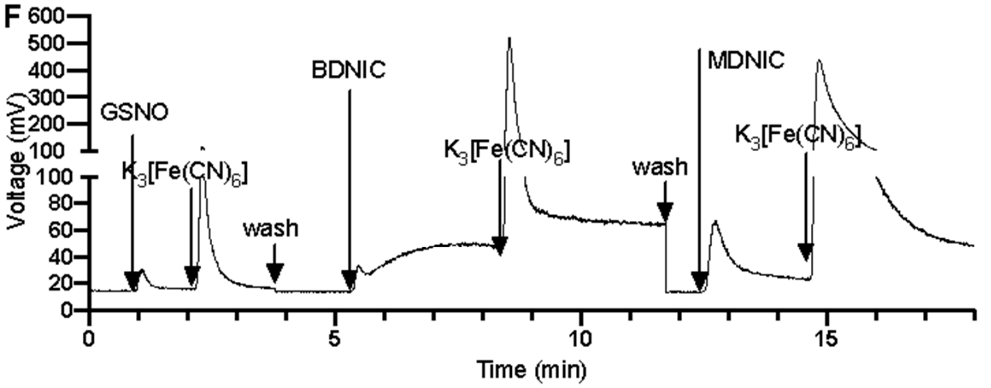
F
GSNO
$K_3[Fe(CN)_6]$
wash
BDNIC
$K_3[Fe(CN)_6]$
wash
MDNIC
$K_3[Fe(CN)_6]$
Voltage (mV)
Time (min)

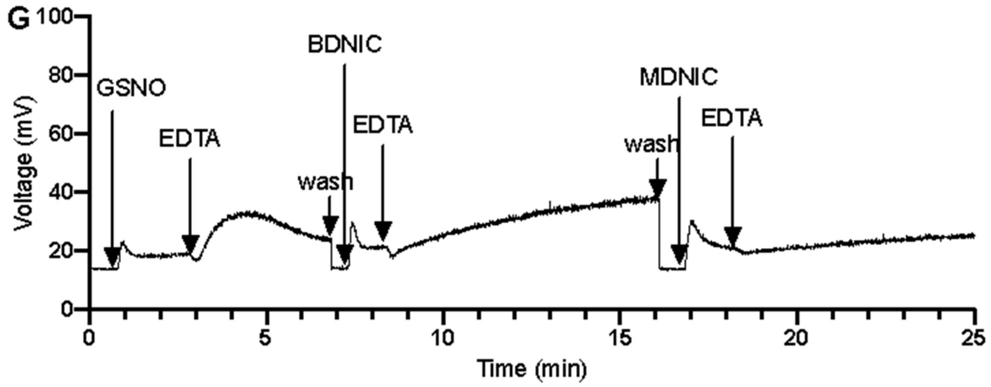
G
GSNO
EDTA
wash
BDNIC
EDTA
wash
MDNIC
EDTA
Voltage (mV)
Time (min)

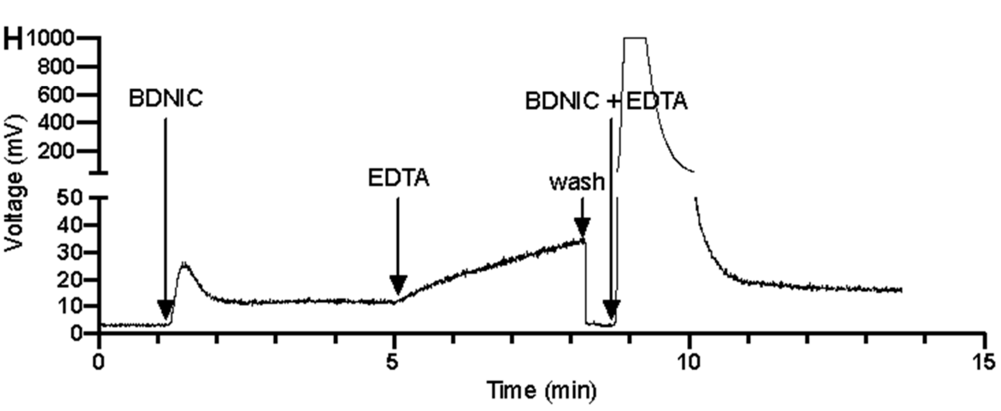
H
BDNIC
EDTA
wash
BDNIC + EDTA
Voltage (mV)
Time (min)

Figure S1. Effects of several chemicals, commonly used in processing of NOx samples, on the stability of GSNO, BDNIC, and MDNIC. n=3. All samples and reagents were injected into a purge vessel (never previously used for I_3^- assay) containing HEPES buffer (no DTPA). Arrows indicate injections or washes. **A)** Representative traces of NO (Proli-NONOate), GSNO, BDNIC, and MDNIC. **B)** Effects of 2.5 mM $HgCl_2$, **C)** Effects of AS (0.125% w/v sulfanilamide in 0.05 N HCl), **D)** Effects of $HgCl_2$+AS, **E)** Effects of 100 µM NEM, **F)** Effects of 0.5 mM ferricyanide, **G-H)** Effects of 25 mM EDTA. All concentrations given above are the final concentrations in the purge vessel. Different from other injections that were directly injected into the purge vessel, the second injection of BDNIC in **(H)** was after its (50 µM) incubation (10 min; room temp in dark) with 500 mM EDTA at a volume ratio of 1:1 in an Eppendorf tube.

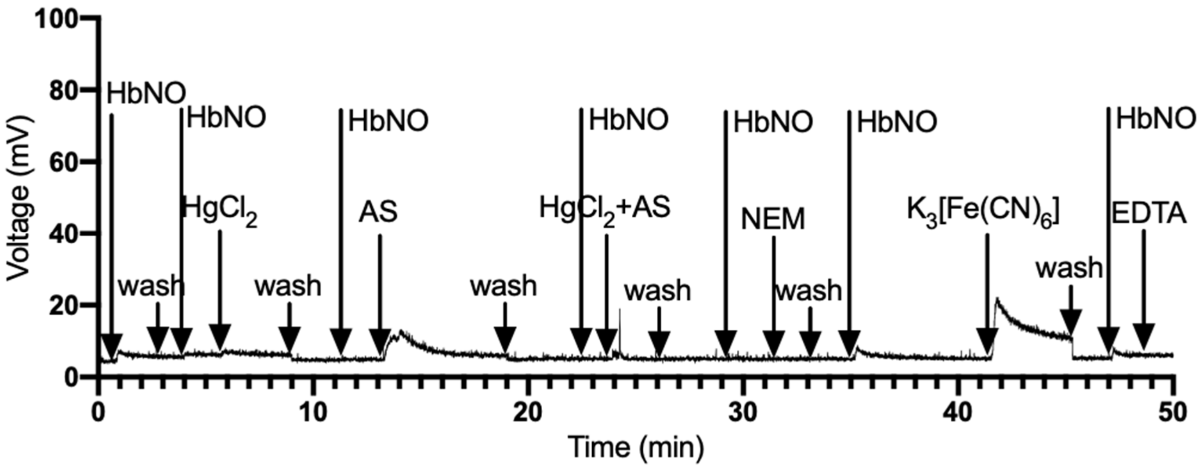

Figure S2. Effects of several chemicals commonly used in processing of NOx samples on the stability of heme-NO (HbNO). n=3. All samples and reagents were injected into a purge vessel (never previously used for I_3^- assay) containing HEPES buffer (no DTPA). Arrows indicate injections or washes. Representative traces of heme-NO (2.5µM), and the effects of 2.5 mM $HgCl_2$, AS (0.125% w/v sulfanilamide in 0.05 N HCl), $HgCl_2$+AS, 100 µM NEM, 0.5 mM ferricyanide, and 25 mM EDTA are shown.

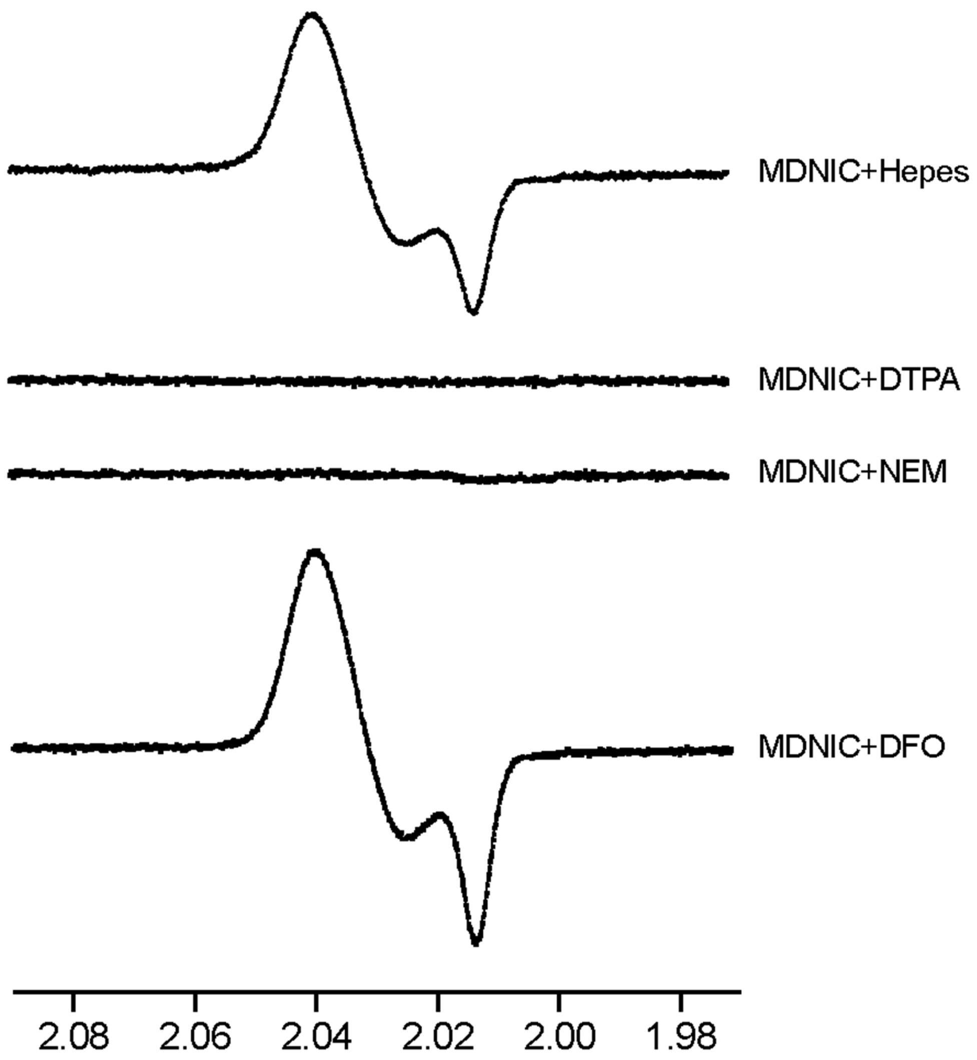

Figure S3. Representative EPR traces showing effects of DTPA, NEM, and DFO on MDNIC signals. n=3. HEPES buffer, 100 μM DTPA, 2 mM NEM, or 1 mM DFO was mixed with 50 μM MDNIC at a volume ratio of 1:1 and measured with EPR at 150 K. All traces were shown in the same scale.

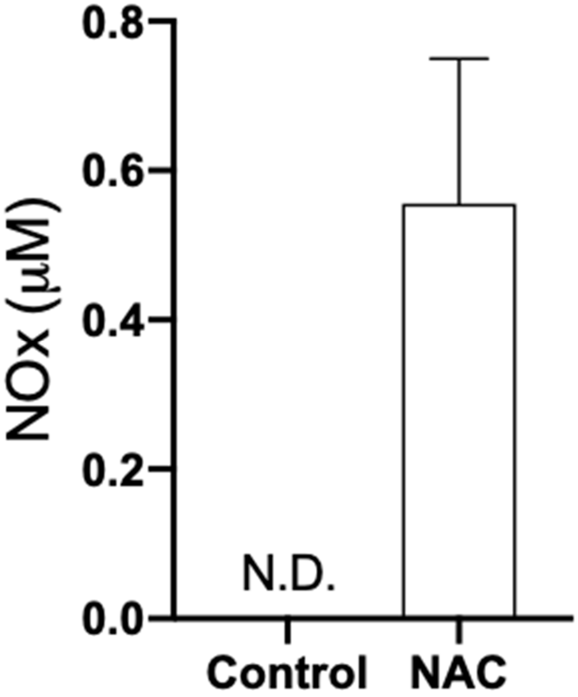

Figure S4. Ferricyanide releases NO from N-acetylcysteine (NAC). n=3. 10 mM of NAC and HEPES buffer (Control) were analyzed with $K_3[Fe(CN)_6]$/PBS assay. Parallel injection of NAC into I_3^- resulted in no detectable NO (not shown).
